# Biophysical informatics reveals distinctive phenotypic signatures and functional diversity of single-cell lineages

**DOI:** 10.1093/bioinformatics/btac833

**Published:** 2022-12-28

**Authors:** Trevor J Chan, Xingjian Zhang, Michael Mak

**Affiliations:** Department of Bioengineering, Yale University, New Haven, CT 06511, USA; Department of Bioengineering, University of Pennsylvania, Philadelphia, PA 19104, USA; Department of Bioengineering, Yale University, New Haven, CT 06511, USA; Department of Bioengineering, Yale University, New Haven, CT 06511, USA

## Abstract

**Motivation:**

In this work, we present an analytical method for quantifying both single-cell morphologies and cell network topologies of tumor cell populations and use it to predict 3D cell behavior.

**Results:**

We utilized a supervised deep learning approach to perform instance segmentation on label-free live cell images across a wide range of cell densities. We measured cell shape properties and characterized network topologies for 136 single-cell clones derived from the YUMM1.7 and YUMMER1.7 mouse melanoma cell lines. Using an unsupervised clustering algorithm, we identified six distinct morphological subclasses. We further observed differences in tumor growth and invasion dynamics across subclasses in an *in vitro* 3D spheroid model. Compared to existing methods for quantifying 2D or 3D phenotype, our analytical method requires less time, needs no specialized equipment and is capable of much higher throughput, making it ideal for applications such as high-throughput drug screening and clinical diagnosis.

**Availability and implementation:**

https://github.com/trevor-chan/Melanoma_NetworkMorphology.

**Supplementary information:**

Supplementary data are available at *Bioinformatics* online.

## 1 Introduction

Cancer cells exhibit a large degree of phenotypic heterogeneity at a range of scales. Primary cancer cells originating from the same cell type but taken from different patients vary drastically in behavior, and even individual cells taken from the same tumor often display differences in gene expression, migratory phenotype, morphology and drug response (Meacham and Morrison, 2013). Cancer cell heterogeneity is observed not only in primary tumors, but in widely studied cancer cell lines such as the MDA-MB-231 breast cancer line (Jonasson *et al.*, 2019).

This variability, which can arise from accumulated genetic and epigenetic mutations (Easwaran *et al.*, 2014; Greaves and Maley, 2012), differentiation of cancer stem cells (Bao *et al.*, 2006; Magee *et al.*, 2012; Mani *et al.*, 2008), volumetric compression in tumor microenvironment (Zhao *et al.*, 2021) and other factors, complicates treatment. Isolated tumor subpopulations have been shown to exhibit varying resistance to drug treatment (Orgogozo *et al.*, 2015) and varying metastatic capability (Balic *et al.*, 2006), contributing to an overall less treatable and more aggressive disease.

Studying cancer cell heterogeneity necessitates observing cells at single-cell resolution. While techniques such as single-cell RNA sequencing are invaluable, limits on sample size, combined with low capture efficiency and high dropout rates, can make scRNA-seq data noisy and difficult to analyze (Buettner *et al.*, 2015; Chen *et al.*, 2019). Furthermore, scRNA-seq fails to capture single-cell phenotype or phenotypic heterogeneity in a population, which significantly limits its usefulness in associating genetic expression with specific cancer phenotypes.

Luckily, directly observing cell phenotype in 2D can provide valuable insight into both the underlying biology and the *in vivo* behavior. Cell morphology arises from a complex interplay of genetics, biochemistry and mechanical interactions. While detailed quantification of morphological features is challenging, recent studies have leveraged informatics approaches to describe and understand physical phenomena (Zhang and Mak, 2021). Specific morphological features on a cellular scale have been linked with underlying cytoskeletal architectures (Christiansen *et al.*, 2018; Ounkomol *et al.*, 2018) and distinct disease phenotypes; morphological features of cancer cells in a clonal population can be used to predict genetic expression (Wu *et al.*, 2020), as well as cell migratory phenotype *in vitro* (Zhang *et al.*, 2021) and metastatic phenotype *in vivo* (Balic *et al.*, 2006).

While these studies base their analysis solely on single-cell morphology, an additional overlooked factor is collective cell behavior. Biological and mechanical interactions between tumor cells, mediated by cell–cell signaling, adhesion and supracellular fluid flow, determine tumor morphology and modes of cell migration (Friedl *et al.*, 2004, 2012; Han *et al.*, 2020; Zhang *et al.*, 2019). Considering the former, the vascular mimicry phenotype in melanoma tumors is overwhelmingly implicated in causing tumor growth, aggressive metastasis and poor survivability (Hendrix *et al.*, 2003).

Understanding cancer behavior on the scale of single cells and the scale of an entire population is essential, but accurately quantifying both cell morphology and collective morphology presents a challenge. Multiple methods exist to quantify morphological characteristics of single cells in 2D, but these methods often require fluorescence labeling and quickly deteriorate in accuracy as cells reach high confluence (Vicar *et al.*, 2019). Thus far, no sufficiently high-throughput quantitative approach has been applied to analyze the collective behavior of cell populations. Here, cell network topology is a less studied metric that efficiently captures key collective behaviors such as cell migration and adhesion (Leggett *et al.*, 2019).

In this work, we quantitatively characterized both single-cell morphologies and cell network topologies of single clonal cell lineages. We utilized a supervised deep learning approach to perform label-free live cell detection and segmentation across a wide range of cell densities. We verified this segmentation method and compared its performance to common unsupervised image segmentation methods for label-free cell images. We measured single-cell morphology and collective cell behavior across a range of densities for 136 single-cell clone (SCC) lineages derived from the closely related YUMM1.7 and YUMMER 1.7 mouse melanoma cell lines (Meeth *et al.*, 2016). Based on an unsupervised hierarchical clustering of these lineages, we identified six distinct morphological subclasses within the YUMM1.7 (YM) and YUMMER1.7 (YMR) SCC lineages. We further demonstrate that cells from different subclasses exhibit distinct and reproducible behavior when embedded as spheroids in a 3D collagen matrix. Drawing on the extensive quantification of 2D and 3D morphological characteristics, we identify a set of strongly correlated (|r|>0.8) variable interactions linking 3D invasive behavior from 2D morphological data and vice versa.

## 2 Results

We developed a pipeline to analyze morphological properties of cell populations from phase contrast image data and to identify distinct morphological classes. These methods comprise three primary components: a segmentation algorithm—for which we implement the Mask R-CNN deep learning architecture; original algorithms for extracting density-dependent cell morphology and network topology properties; and a hierarchical clustering algorithm for subclass identification.

### 2.1 Image segmentation

We seek to characterize both single-cell and cell population morphologies. Characterization of single-cell morphology alone is common and relatively straightforward; it requires imaging of sparse cells at a single time point. Acquiring these images, whether in brightfield or using fluorescent labeling, can be accomplished using a number of conventional methods. Characterization of cell population morphology is much more complex. For one, as population morphology is highly dependent on cell density, imaging populations over multiple days is a necessity. Further, as cell populations develop complex morphologies only at mid to high densities where cells are in contact with each other, the problem of achieving accurate single-cell segmentation of images becomes much more difficult.

**Fig. 1.**
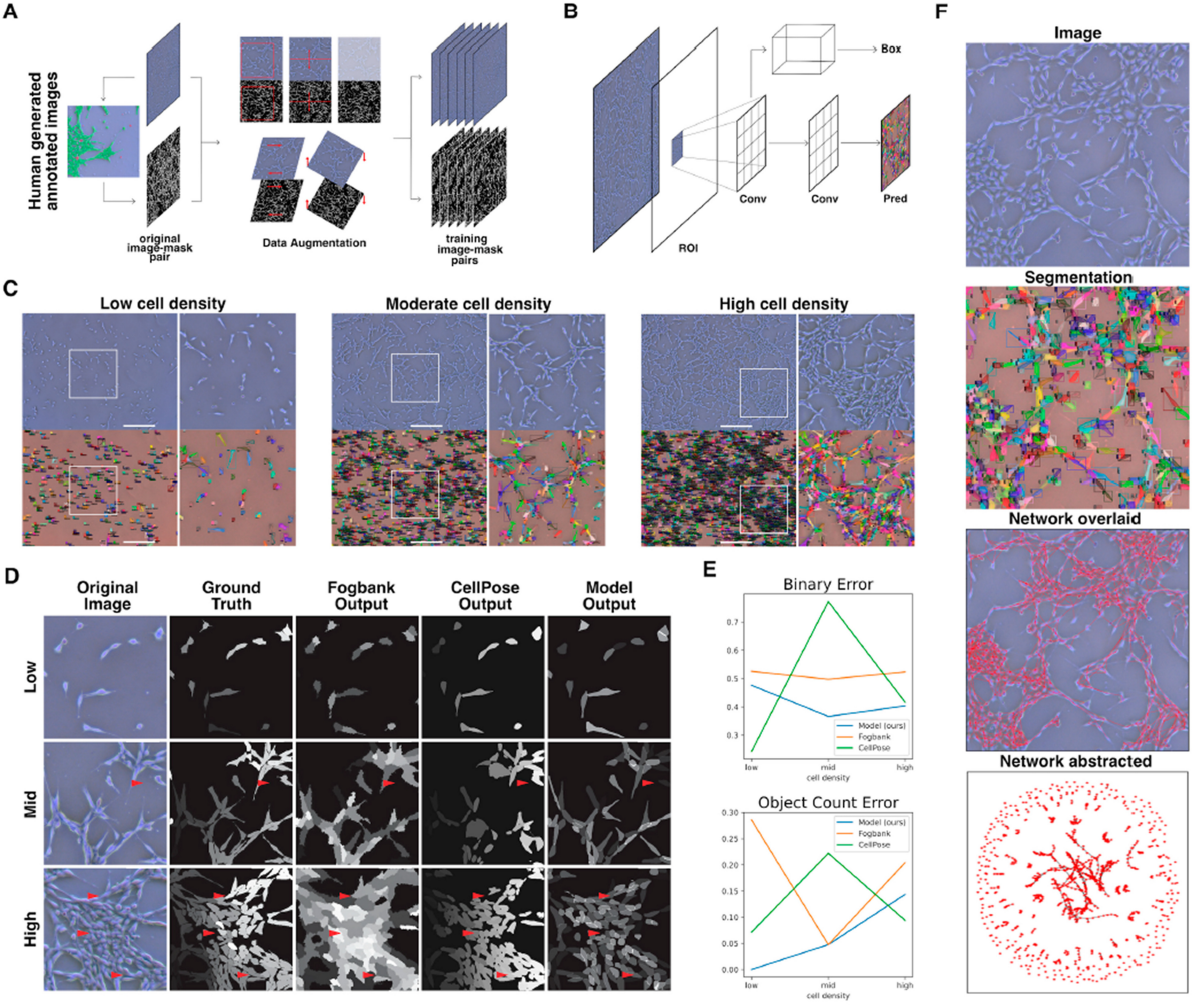
Instance segmentation of phase contrast images was performed using a convolutional neural net. (**A**) Training data were prepared in two steps: first, segments of phase contrast images were annotated manually, producing an original mask from an original image. Second, a series of random augmentations—rotation, shear, contrast, reflection and crop—were applied to each original image-mask pair to generate multiple augmented images-mask pairs from each original. The model was trained on the resulting augmented dataset. (**B**) The Mask RCNN architecture was trained and used to perform instance segmentation. This modular architecture uses a classifier to detect individual objects in an image and produces a binary mask for each. For more details, see the paper: https://arxiv.org/abs/1703.06870. (**C**) Representative ground truth images and segmentation visuals for a range of cell densities. Individual cells are marked in the segmentation visuals with a unique color bounding box and mask overlay, as well as a classification confidence score (0.00–1.00). We demonstrate the model’s ability to draw accurate masks and distinguish between adjacent cells. Scale bar is 500 microns. (**D**) We compared our model outputs against commonly used unsupervised and supervised segmentation methods Fogbank and CellPose. We found that the model more closely and consistently matched human-generated ground truth image segmentations across image cell densities. At mid- and high-cell density, the learned model demonstrated much higher accuracy at separating closely adjacent cells and identifying small gaps between densely packed cells. Scale bar is 100 microns. (**E**) We used two metrics to compare segmentation accuracy: object count error, related to individual object detection, and binary error, related to foreground segmentation. Our model shows consistently high performance across the full range of cell confluencies compared to both Fogbank and CellPose. (**F**) Image segmentations are used to obtain single-cell morphology and cell network topologies. Shape properties, including area, circularity and aspect ratio, are derived from individual cell masks (second from top). A network is constructed from cell positions; individual cells constitute nodes of a graph and edges are drawn between adjacent cells (second from bottom). Once generated, the network can be represented irrespective of cell positions (bottom).

We address these challenges by using a deep learning algorithm to perform segmentation on brightfield images. This method brings the dual advantages of accurate segmentation even at very high-cell densities (>80% cell confluency) and eliminating the risk of adverse effects from fluorescent cell labeling over long periods of time. We implement the previously described Mask R-CNN architecture (Fig. 1b) to perform instance segmentation on individual cells (He *et al.*, 2017). A training dataset comprising 5000 cell masks was produced manually from dozens of images across a range of cell confluencies and augmented at training (Fig. 1a). A novel non-max-suppression algorithm was used to prune duplicate cell predictions. Model performance is validated by comparing model outputs with human-annotated images (considered ground truth) on unseen images (Fig. 1c). We further compare the model performance to two existing methods, FogBank and CellPose. FogBank is a commonly used all-in-one image segmentation and cell separation pipeline for label-free cell images (Schüffler *et al.*, 2015). The choice of FogBank was based on findings by Vicar *et al.* (2019) that compared it favorably against a number of common contrast microscopy segmentation methods. CellPose is a supervised algorithm for cell segmentation trained on a wide array of community-supplied fluorescent and non-fluorescent microscopy images (Pachitariu and Stringer, 2022; Stringer *et al.*, 2021). For comparison, we define two metrics of performance: binary error, $e_{\text{binary}}=|A_{\text{gt}}-A_{\text{pred}}|/A_{\text{gt}}$, where *A*_gt_ and *A*_pred_ are the foreground area of the ground truth segmentation and predicted segmentation, respectively, and object count error, $e_{\text{object}}=|N_{\text{gt}}-N_{\text{pred}}|/N_{\text{gt}}$, where *N*_gt_ and *N*_pred_ are the number of cells in the ground truth segmentation and predicted segmentation, respectively. We found that the learned model outperformed the FogBank algorithm in both metrics at varying densities (Fig. 1e), and achieved greater accuracy when distinguishing between closely adjacent cells in images with high-cell densities (Fig. 1d). CellPose (CellPose v2, trained on community images) was capable of generally high performance, on par with our model in segmenting low-density and high-density images. However, it fell short when segmenting images of middling cell density. Here, it often missed cells and predicted empty gaps between cells to be cells (Fig. 1d). Achieving optimal performance for each cell density also required changing the model sensitivity parameters, so we could not use the same settings for every test image. Instructions and code for training and implementing these models are made publicly available at https://github.com/trevor-chan/Melanoma\_NetworkMorphology (see Materials and Methods section in Supporting Information for additional details).

### 2.2 Isolating clonal populations

**Fig. 2.**
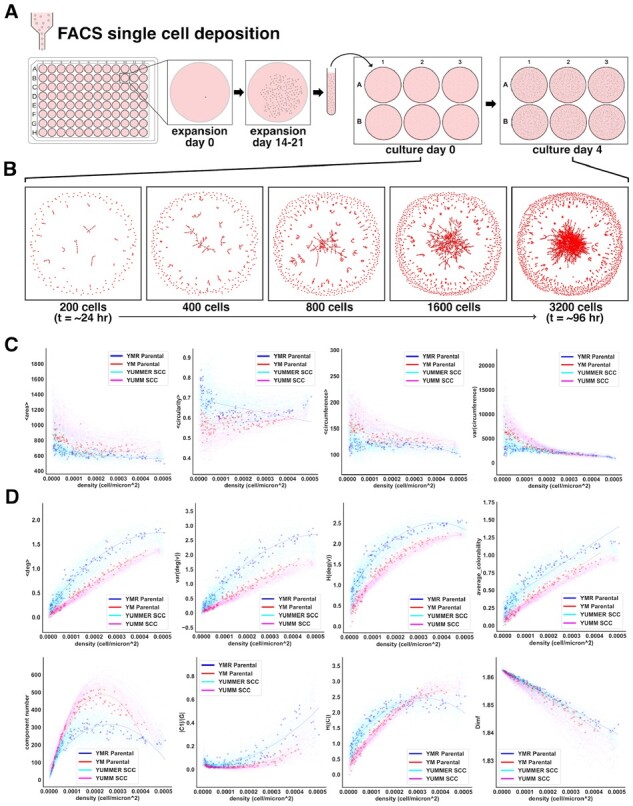
One hundred thirty-six SCC lineages are characterized by single-cell morphology and cell network morphology over time. (**A**) Single-cell clone lines are obtained through FACS deposition of one cell into each well of a 96-well plate. Following an initial expansion period of 14–21 days, cell clones are trypsinized, suspended, and re-seeded at low density into wells of a 6-well plate. Cells expand and are imaged over the following 96 h. (**B**) Generated cell networks (abstracted from position) represent a typical growth pattern of a clone line over the second expansion period. Cell populations are initially sparsely seeded with few adjacent cells. Cell number grows exponentially and cell–cell adjacencies become more common as cells pack more densely, resulting in larger and more connected cell clusters. In this analysis, density, not time, is the primary independent variable. Slight differences in initial cell density and propagation rate translate into high variance in measured morphological properties at later time points. (**C** and **D**) Cell shape properties (**C**) and network topology-derived properties (**D**) describe complex patterns of growth when plotted against cell density. Here, YM and YMR parental and clonal lineages are plotted. Each point corresponds to a single image and contains data corresponding to the derived cell network and hundreds to thousands of cells. The collection of all data points from one clone over the course of a 4-day expansion period constitutes a unique morphological progression. We use polynomial regression to fit curves to each clone morphological progression.

We generate SCCs by using flow cytometry sorting to deposit single cells into wells of 96-well plates. We expand each clone until cells reach 90% confluence, at which point cell populations are trypsinized and seeded at low confluency into wells of a 6-well plate for imaging (Fig. 2a). Images are taken on a Leica DMi1 phase contrast microscope at 5× magnification with a 0.12 numerical aperture. Images are captured over one 4-day subculture cycle.

### 2.3 Description and derivation of single-cell and network variables

From segmented images, we derived a number of common single-cell morphological properties. In Figure 2c, we show that cell shape depends greatly on cell confluency. As cells pack more densely, cell area decreases and, at very high densities, circularity increases. We also observed that distinct clonal populations exhibited a range of single-cell morphologies.

Notably, genetically identical cells within a clonal population exhibited substantial morphological variation, depicted here as property variance. Furthermore, the degree of variance differed from clonal lineage to clonal lineage (Fig. 2c.4 and d.2). Morphological variability in genetically uniform populations has been shown to correlate with increased transcriptomic and phenotypic variability, and overall higher metastatic capability *in vivo* (Nguyen *et al.*, 2016). Here, clonal populations exhibiting abnormally high intrinsic morphological variance might be prime candidates for further research into cancer stem cell-like behavior.

In addition to quantifying single-cell morphology, we quantified features of cell population networks generated from segmented images. Every cell in the segmentation output corresponds to a node in the graph, and edges are constructed between adjacent cells, here defined as two cells with a minimum edge-to-edge distance <5 microns (Fig. 1f). From the resulting network, we derived several relevant topological parameters that indirectly describe cell adhesion, migration and clustering characteristics (Fig. 2d).

Average degree (<deg>), as well as the closely associated variance in vertex degree (var(deg(v))) and vertex degree entropy (H(deg(v))), describes the likelihood of cell–cell adjacencies in a network. We calculated the Shannon entropy of a property $H(x)=-\sum_{i=1}^{n}P(x_{i})ln(P(x_{i}))$ where $P(x_{i})$ is the probability of a single measurement of that property. Graph colorability—taken here as an average over all components of the graph (average colorability) $<\chi (G)>:=\sum_{i=1}^{n}\chi (c_{i})/n$ with *c_i_* as the chromatic number of a single component of the graph—best describes the maximum achievable density of cells in a graph. Graph components offer another useful window into population behavior by clustering the network into groups of connected cells. We look at the number of discrete components in a network (component number), the entropy of the component vertex mass distribution H(|C|), and the relative vertex mass of the single largest component $\left|c_{1}|/|G|=|c_{1}|/\sum_{i=1}^{n}|c_{i}\right|$ where $\left|c_{i}\right|$ denotes the mass of a component *c_i_* and *c*_1_ is the largest component of the network. Additionally, the fractal dimension (Dimf) of a cell network, $dim_{\mathrm{box}}(S):=\underset{\epsilon \to 0}{\lim}\frac{\;\text{log}\;N(\epsilon )}{\;\text{log}\;1/\epsilon }$ Where *S* is a network in 2D and N(ϵ) is the number of boxes of side length required to cover *S*, provides a metric through which to evaluate network space filling behavior. We track these properties over a full subculture cycle and perform polynomial regression to approximate density-dependent trends in network structure (see Supplementary Fig. S1 for a full list of derived variables). See also Supporting Information, Additional Results.

Lastly, we performed additional experiments to verify the stability of these traits over a period of multiple weeks. These results are elaborated in Supplementary Section S1B, Supporting Information, Additional Results, Temporal stability of SCC morphological profiles section and Supplementary Fig. S6.

### 2.4 Clustering of single-cell clones

**Fig. 3.**
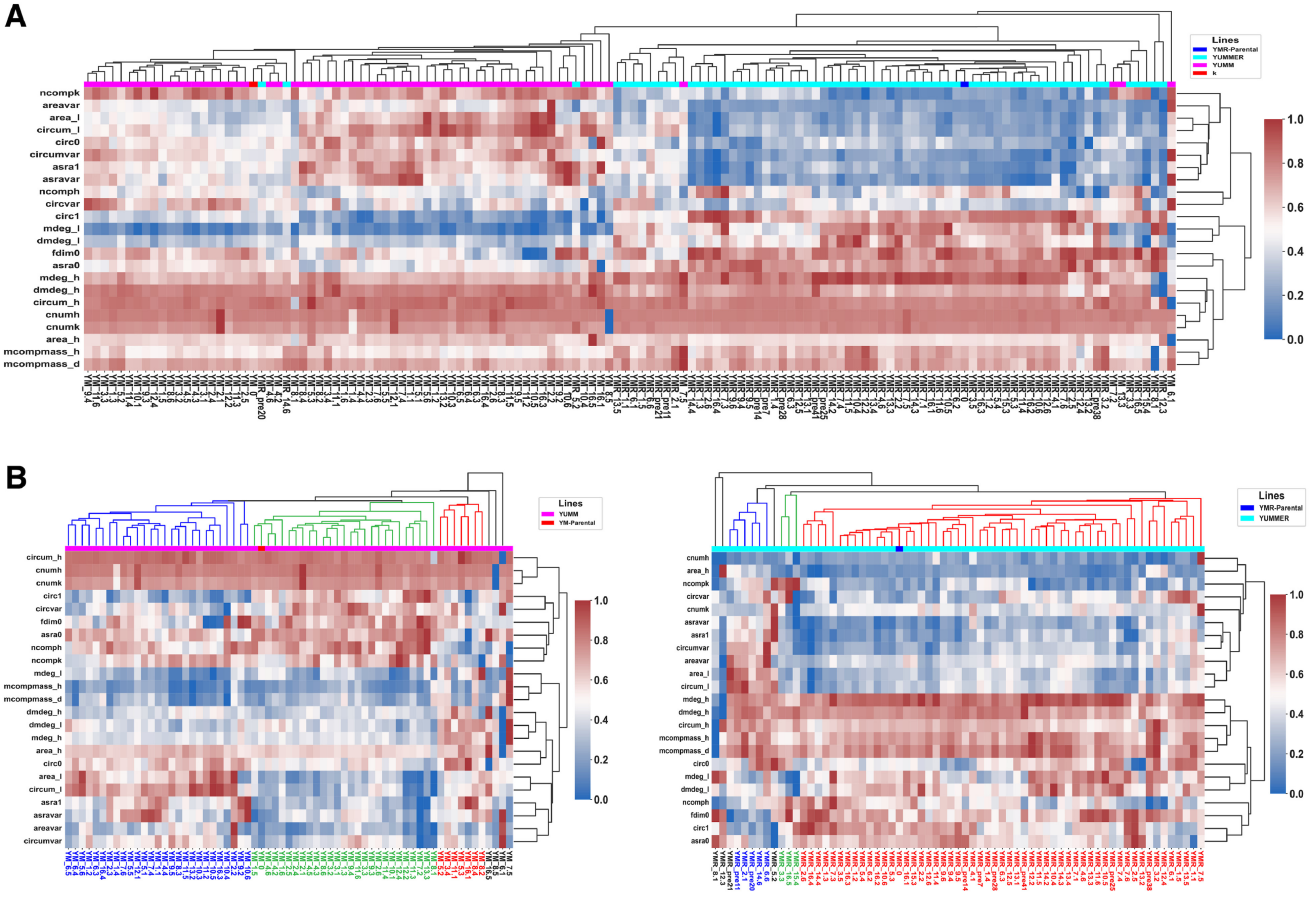
Clustering of SCC lineages distinguishes between YM and YMR clones and identifies six morphological subclasses of interest. (**A**) Hierarchical clustering of the 136 clone lineages robustly distinguishes between YM-derived and YMR-derived clones. (**B**) Dividing the clones into their respective cell lines, further hierarchical clustering identifies six morphological subclasses, three within YM and three within YMR.

Clonal lineages were characterized by a set of morphological growth variables, which summarize qualities of single-cell shape and of network structure for the full range of one subculture cycle. These were obtained from the single-cell and cell network property polynomial regressions. Using these growth variables, we performed agglomerative hierarchical clustering on all SCC populations. As the YUMMER cell line is itself a genetically distinct subpopulation of YUMM expanded from a UV-irradiated SCC (Wang *et al.*, 2017), we expected to see morphological differences between these lines, and this expectation was borne out in both parental and clonal daughter populations. With few exceptions, YUMM-derived clones and YUMMER-derived clones constituted two superclasses in the clustering hierarchy (Fig. 3a, Supplementary Fig. S2). Looking at the hierarchical clustering dendrogram, we can identify a subset of morphological growth variables that are strong predictors of YM vs. YMR superclass affiliation. These include the topological parameters average degree (mdeg_l) and network component number (ncompk), as well as the single-cell morphological parameters circularity (circ1), and variance in area (areavar).

We also observed that the set of variables that predict cell line were not the same as the set of variables determining intra-cell-line subclass affiliation (Fig. 3a and b). This may indicate large differences in behavior between YM and YMR cell lines not captured by morphological features alone. Based on this observation, we divide the SCCs into YUMM-derived clones and the YUMMER-derived clones and perform hierarchical clustering on each group, resulting in two new hierarchical dendrograms. Separating the analysis here allows for better resolution when identifying subclasses and when identifying strongly predictive morphological growth variables. Specifying a horizontal cut across each dendrogram divides populations into three distinct subclasses for each cell line (Fig. 3b). We verified the subgroup selection using UMAP clustering and observed a similar grouping of clonal lineages according to cell line (Supplementary Figs S2 and S3) and morphological subclass (Supplementary Figs S2 and S4). We also show representative images of a cell network for each subclass taken at moderate confluency (Supplementary Fig. S2), and a UMAP clustering of all networks with the selected representative lines highlighted (Supplementary Fig. S4e and f).

### 2.5 3D spheroid formation and invasion

**Fig. 4.**
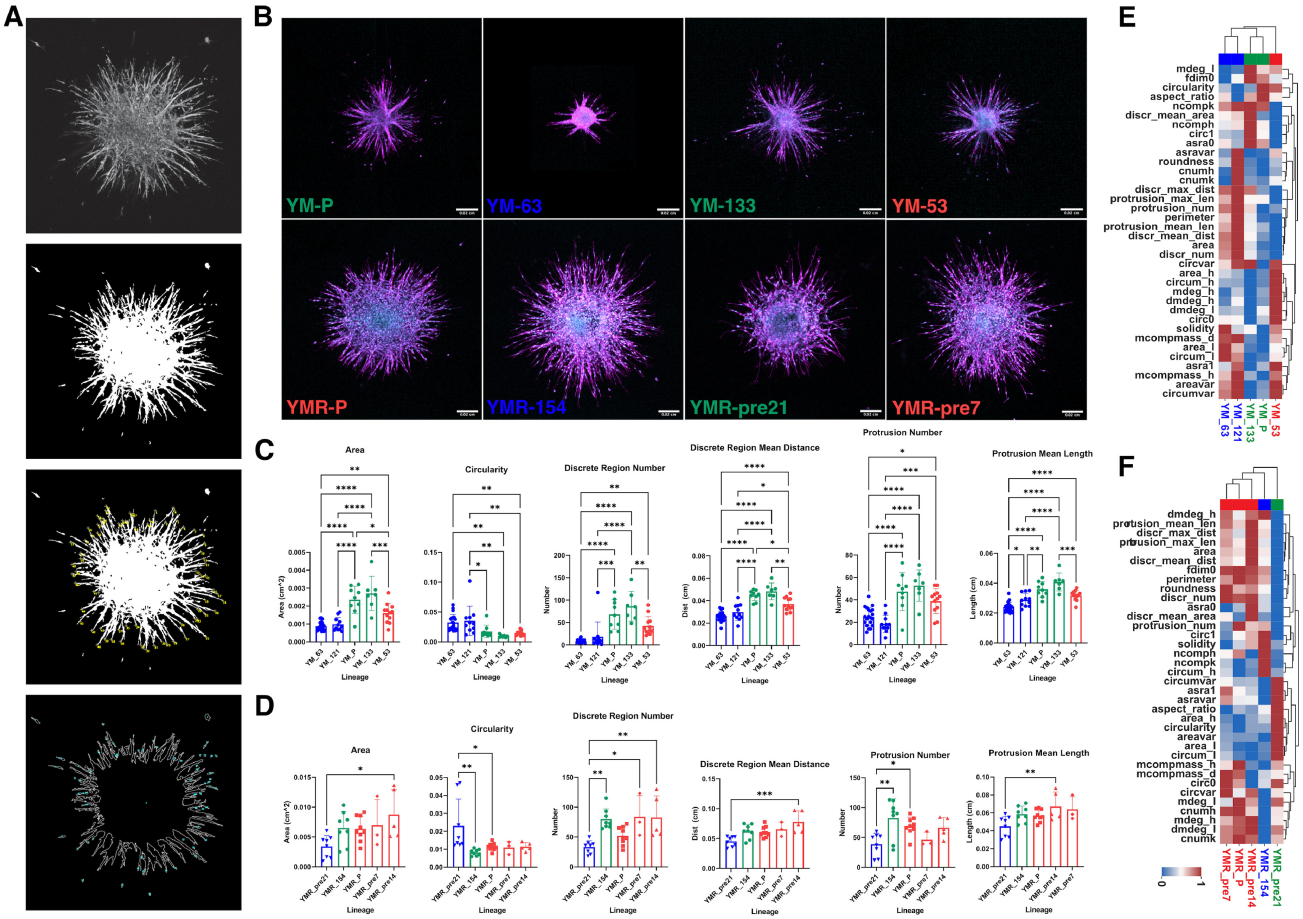
2D-derived morphological subclass predicts 3D spheroid invasiveness. (**A**) Spheroid shape and invasiveness are measured from projected, binarized, z-stacks. Confocal imaging of the entire spheroid yields z-slices in 5-micron height increments. Fifteen slices, over 75 microns, are selected from the center of each spheroid and max projected to create one image (far left), which is then binarized (center left). From the binarized image, shape properties are derived, spheroid protrusions are counted and measured (center right) and disseminated cells are counted and measured (far right). (**B**) Representative images of spheroids generated from parental and clonal lineages embedded in a 3D collagen gel show vast differences in cell migratory phenotype. F-actin (magenta, peripheral) and DNA (cyan, central) are labeled. Scale bar is 200 microns. (**C** and **D**) We compare the 3D shape and invasive behavior of subclass representative clones with each other. Statistics for comparisons are performed using a one-way ANOVA test and *post hoc* Tukey for each 3D parameter. Significant differences between spheroid shape (area, circularity) and invasion (protrusion and disseminated cell dynamics) exist between populations across subclasses and are much weaker for populations within the same subclass. (*, **, ***, **** correspond to *P* < 0.05, 0.01, 0.001, 0.0001, respectively). (**E** and **F**) Repeating the hierarchical clustering from earlier now including 3D spheroid parameters alongside 2D cell shape and network topology parameters reiterates the clustering obtained from only 2D parameters. (A color version of this figure appears in the online version of this article)

We further investigate the behavior and morphology of these derived subclasses in a 3D assay. We select 1–2 representative clonal lineages for each of the identified subclasses from which to generate spheroids (clusters of 1000 cells) that are subsequently embedded into collagen matrices. We image the spheroid and the surrounding matrix in brightfield and reflectance over 72 h before fixing and staining the cultures for actin and cell nuclei and imaging using confocal microscopy. In order to derive quantitative measurements from spheroid immunofluorescence (IF) images, we select a 75 micron wide stack of actin fluorescence images from the middle of each spheroid and project it along the *z*-axis (Fig. 4b, Supplementary Videos S1–S10). We then binarize the projected image and calculate shape properties of the spheroid, mainly: area, circularity, and solidity (Fig. 4a). We also count the number of regions that are not connected to the spheroid through the actin channel. We consider these to be single cells or groups of cells that have migrated away from the spheroid. For each of these regions, we calculate their area and their centroid-to-centroid distance from the spheroid. Additionally, we count the number of protrusions emitting from the spheroid and record the length of each, defined here as the distance from protrusion tip to spheroid centroid. We show that there are significant differences in spheroid area, the number and distance of disconnected regions, and the number and length of protrusions between representative clone lineages derived from YM and YMR cells (Fig. 4c, Supplementary Fig. S5).

Furthermore, we observe similar 3D spheroid morphologies within subclasses of SCC lineages and distinct morphologies across subclasses. Statistical differences in spheroid area, circularity, cell invasion (distance and number) and protrusion formation (length and number) are small between populations within a subclass and orders of magnitude greater for populations belonging to different subclasses (Fig. 4c and d). Differences across subclasses are most apparent when looking at metrics of cell invasion, specifically the number and length of spheroid protrusions and the number and distance of disconnected migrating cells. These findings demonstrate that a hierarchical clustering of lineages based solely on the described 2D analysis can also describe cell invasive behavior in 3D, and they indicate that the same underlying cellular mechanisms dictating single-cell morphology and cell network formation in 2D also dictate cell morphology and invasion in 3D.

Having characterized 10-cell lineages (8 representative clonal lineages and 2 parental lines) both in 2D and 3D, we repeat the hierarchical clustering of these lineages considering both 2D cell morphology and network topology, as well as 3D spheroid shape and invasion characteristics. The added 3D variables contribute significantly to the updated clustering and recapitulate the results of the 2D clustering; representative lineages are clustered into the same subclasses and relations between these subclasses are preserved.

### 2.6 2D and 3D variable correlation analysis

**Fig. 5.**
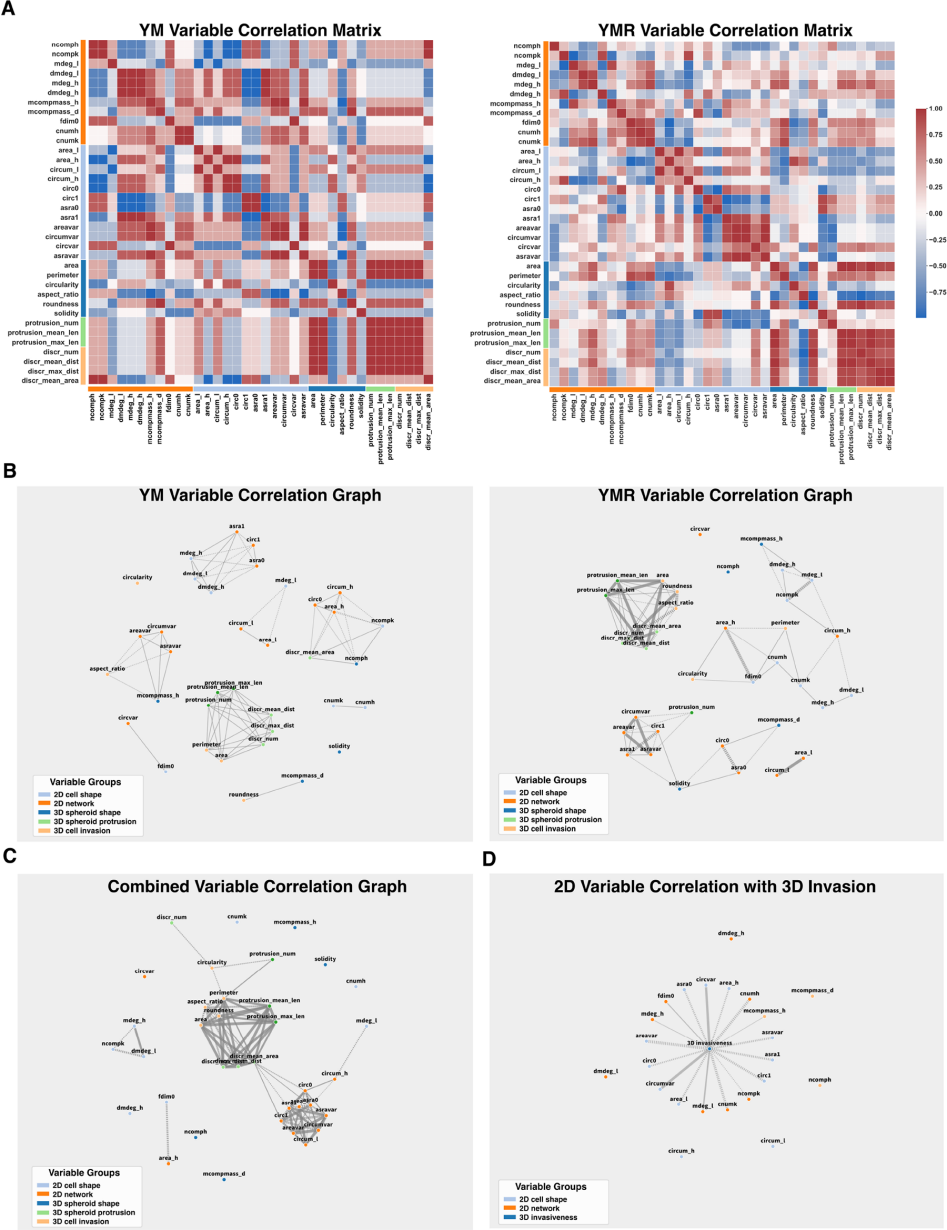
Multiple strong correlations exist between 2D cell shape variables, 2D network topology variables, 3D spheroid shape variables and 3D spheroid invasion variables. (**A**) Correlation matrices show numerous strong positive and negative correlations between morphological variables. Correlations are common between variables describing behavior at the same scale (single-cell scale, cell network scale and cell spheroid scale) but also exist between variables describing behavior across scales. (**B**–**D**) A variable correlation graph provides an alternate visualization of variable relationships. Here, nodes of the graph represent variables and are colored according to the scale of behavior each describes—the variable group. Solid edges represent positive correlations and dashed edges represent negative correlations. Thicker lines represent stronger (larger magnitude) correlations. (B) Variable correlation graphs depict YM and YMR SCC populations separately. Correlation matrices Edges represent a variable to variable Spearman correlation coefficient magnitude >0.8. (C) Variable correlation graph depicts YM and YMR SCC populations together. Correlation matrices Edges represent a variable to variable Spearman correlation coefficient magnitude >0.8. (D) Variable correlation graph depicts the correlations between 2D variables and a single variable for 3D invasiveness only. (Relations between 2D variables are not drawn.) This graph includes SCC populations from both the YM and YMR cell lines. Correlation matrices Edges represent a variable to variable Spearman correlation coefficient magnitude >0.3. See also Supplementary Fig. S7.

Quantitative characterization of these lineages in both 2D and 3D allows us to identify a number of positive and negative correlations between morphological variables. For each of YUMM and YUMMER clones, we calculate the correlation matrix for 2D and 3D variables (Fig. 5a). Correlation coefficients are calculated using a Spearman correlation test. To better understand variable relationships, we break variables into five variable groups describing 2D cell network topology (11 variables), 2D single-cell morphology (12 variables), 3D spheroid shape (6 variables), 3D spheroid protrusions (3 variables) and 3D spheroid disseminated cells (4 variables).

As expected, we show abundant strong positive or negative correlations between variables belonging to the same variable group. These often describe similar cell characteristics or behaviors, for instance cell area and cell circumference in 2D. We also find a number of strong correlations between variables across variable groups. In particular, metrics of collective behavior, including fractal dimension (fdim0) and mean component mass (mcompmass_h), correlate with cell circularity, area and circumference.

We construct a variable graph depicting only very strong positive or negative correlations (|r|>0.8) between variables for both the YM and YMR cell lines (Fig. 5b). We also perform a combined analysis on SCCs from YM and YMR and identify a set of shared morphological features that predict 3D behavior. We plot a variable graph depicting strong correlations (|r|>0.8) between variables for the combined populations (Fig. 5c). Strong correlations exist for both YM and YMR populations, indicating overlap in the mechanisms determining cell morphology and collective behavior in 2D and 3D between the two distinct cell lines. However, the variable correlation matrices for the YM-derived and YMR-derived populations are not identical, suggesting the presence of secondary mechanisms contributing to morphological and collective behavior that are not common between the two cell lines. Lastly, we normalize and average 6 3D metrics indicative of high spheroid invasion, namely protrusion number (*n_p_*), protrusion mean (avg_*p*_) and maximum (max_*p*_) length, discrete invaded cell number (*n_c_*) and discrete invaded cell mean (avg_*c*_) and maximum (max_*c*_) distance, into one comprehensive metric for 3D invasiveness, ${\text{invasion}}_{3D}:=(\bar{n_{p}}+\bar{n_{c}}+\bar{{\text{avg}}_{p}}+\bar{{\text{avg}}_{c}}+\bar{{\text{max}}_{p}}+\bar{{\text{max}}_{c}})/6$ with $\bar{x}$ denoting a property normalized between 0 and 1, where values of 0 and 1 indicate the minimum and maximum measured value of that property over the relevant cell lines. We plot variable correlations between this 3D invasion metric and all described 2D variables (Fig. 5d). This distilled information allows us to estimate the bulk 3D invasiveness of a clonal population from the 2D analysis.

## 3 Discussion

Cancer cells exhibit a large degree of phenotypic heterogeneity, in part due to elevated epigenetic and genetic instability. In these cases, accumulated differences in genetic expression affect cell behavior, including in cell morphology, cell migration and collective cell behavior (Sun and Yu, 2015; Liu *et al.*, 2019). Previous studies have looked at variation in cell morphology in cell lines and primary tumor populations, but often fall short of robustly quantifying both cell morphology and collective cell behavior. On the other hand, multiple works examining biological jamming and unjamming phase transitions have quantified collective cell behavior in depth, but these methods rely on high temporal resolution dynamic information that makes them difficult to replicate at large scale (Kang *et al.*, 2021; Yang *et al.*, 2021).

Past research has highlighted the value of 3D cell culture methods that better resemble a realistic *in vivo* environment compared to traditional 2D methods (Duval *et al.*, 2017; Jensen and Teng, 2020). 3D methods are especially crucial in the context of studying cell migration, adhesion, and cell–cell interactions, as cells can rely on different biochemical and mechanical mechanisms in 3D and 2D (Fraley *et al.*, 2010). A consequence of this is the often unintuitive connections between observed 2D phenotypes and 3D phenotypes of cells. For example, cell migration speed in 2D does not correlate with invasion in 3D, but 2D cell morphology often does (Baskaran *et al.*, 2020). Nonetheless, 2D cell phenotype as a whole is shown to correlate with clinical outcomes (Nguyen *et al.*, 2016; Wu *et al.*, 2020), and we demonstrate that both cell 2D phenotype and cell network structure correlate with 3D invasion, prompting the question of what mechanisms underlie this correlation.

Of particular interest to us are gene expression pathways mediating cellular adhesion and signaling, many of which are also implicated in the epithelial to mesenchymal transition (EMT). Prior studies have shown that EMT signaling, inducing a full or partial mesenchymal transition, is a primary determinant of cell migration mode (Gonzalez and Medici, 2014). It affects the expression of focal adhesion proteins and the regulation of actin dynamics. The former is crucial both for cell spreading on stiff 2D substrates and cell 3D motility by regulating single-cell protrusions (Fraley *et al.*, 2010). The latter affects cell protrusions in 2D and motility and matrix remodeling in 3D (Malandrino *et al.*, 2019; Meyer *et al.*, 2012). Intracellular and intercellular signaling influence cell networks in 2D and 3D primarily through the modulation of epithelial-like behavior. Paracrine signaling contributes to 2D morphology and 3D migration speed in a collagen matrix (Meyer *et al.*, 2012). Additionally, various modes of collective cell migration depend heavily on intercellular adhesion proteins (Friedl and Mayor, 2017). E-cadherin, N-cadherin and *β*-catenin are among molecules known to affect various aspects of cell metastasis, including the mode of collective migration and the survivability of the metastasizing cells (Ilina *et al.*, 2020; Padmanaban *et al.*, 2019). Taken collectively, differential expression of these key proteins regulating intercellular signaling, cellular adhesion to substrate or matrix and intercellular adhesion, result in distinct and recognizable profiles of cellular morphology and behavior in both 2D and 3D.

Within this framework of differential gene expression, differences between the clonal lineages studied here can be understood as elevated or decreased levels of cell–substrate adhesion proteins, cell–cell adhesions and intracellular signaling molecules. Stark distinctions, such as those between YUMM-derived lineages and YUMMER-derived lineages, are more likely to be due to genetic mutations, while subtler differences, such as those between intra-cell-line subclasses, may be primarily epigenetic changes, arising from inherent inhomogeneities already present in the cell line, genetic instability, or differences in initial growth conditions, but nonetheless persisting for many generations (Liu *et al.*, 2019).

In our analysis, we observed specific 2D behaviors indicative of altered signaling and adhesion dynamics and found correlated growth and invasion behaviors in 3D. Strong cell–cell adhesion, measured by average network degree and network component mass, corresponded to high 3D invasiveness. At the single-cell level, high variance in cell shape, specifically circumference and circularity, also correlated with increased 3D invasiveness, indicating that phenotypic heterogeneity aids cell invasion.

While more experiments are required to determine the link between *in vitro* invasiveness and *in vivo* disease progression, these methods introduce the possibility of estimating how tumor cell populations will behave *in vivo* without the need for the more complex and extensive 3D culture experiments. Such an approach would be immediately useful to the area of high-throughput screening for new drugs. Drug screening lends itself especially well to automation and has been slow to fully incorporate 3D disease models, despite significant predicted gains in screening precision (Langhans, 2018). Here, 2D cell phenotype can serve as a valuable metric by which to identify promising compounds for further 3D and animal model studies.

## 4 Conclusion

We present a promising analytical approach to studying cell phenotype in 2D and 3D. We utilize a deep learning segmentation algorithm applied to phase contrast cell images and describe a new comprehensive analysis of 2D cell morphology and network topology. We further demonstrate that the 2D analysis is able to discriminate between the similar YUMM and YUMMER cell lines and to identify morphological subclasses within each cell line. We then show that the subclass clustering resulting from the 2D analysis accurately predicts spheroid growth and cell invasion in a 3D collagen environment. Lastly, we identify variable relationships between 2D and 3D variable groups present in either or both of the YUMM and YUMMER populations.

Compared to established protocols for 3D spheroid culture, our 2D analytical method is faster (taking days as opposed to weeks), is orders of magnitude higher throughput (including millions of cells as opposed to thousands, hundreds of (sub)populations as opposed to dozens), and requires no specialized culture or imaging equipment (normal tissue culture plates and brightfield or phase contrast microscopy, as opposed to 3D culture and confocal microscopy). Most importantly, our method demonstrates a high ability to distinguish between populations with high and low invasiveness. The potential applications of our methods are abundant, ranging from high-throughput drug screening to low-cost clinical diagnosis.

## Materials and Methods

Materials and Methods can be found in Supporting Information.

## Supplementary Material

btac833_Supplementary_Data

## Data Availability

Code used in this article is publicly available at https://github.com/trevor-chan/Melanoma\_NetworkMorphology. Relevant data can be provided by TC pending a completed material transfer agreement. Requests for the data or code should be submitted to: tjchan@seas.upenn.edu.
