# Supplementary material for: Biophysical informatics reveals distinctive phenotypic signatures and functional diversity of single-cell lineages
Source: Bioinformatics. 2022 Dec 28;39(1):btac833. doi: 10.1093/bioinformatics/btac833 (PMC9825265; doi:10.1093/bioinformatics/btac833)
Supplement: btac833_Supplementary_Data [file btac833_supplementary_data.pdf]

Bioinformatics, 2022, 1–12
doi: DOI HERE
Advance Access Publication Date: Day Month Year
Paper

Supporting Information: Biophysical informatics reveals distinctive phenotypic signatures and functional diversity of single cell lineages

Trevor J. Chan,[1,2] Xingjian Zhang[1] and Michael Mak[1,*]

[1]Bioengineering, Yale University, 55 Prospect St, 06511, CT, United States and [2]Bioengineering, University of Pennsylvania, 3400 Spruce St, 19104, PA, United States

*Corresponding author. michael.mak@yale.edu

Abstract

Additional Results

Density Dependence of Cell Morphology and Cell Network Structure

Both the cell morphological properties and network topologies of an SCC change drastically and nonlinearly as the density of cells increases. SCC averages across a handful of typical shape properties span a wide range of values at low cell densities and gradually converge at higher densities, likely due in part to space limitations forcing cells to adopt similar shapes (fig 2c). We note that previous studies focusing only on single cell morphology do not rigorously consider cell density, a potentially significant factor, when deriving shape or variance in shape parameters.

As expected, topologies of cell networks are even more closely dependent on cell density (fig 2d). At very low density (sparse cell seeding), networks of all lineages consist of disconnected nodes and are essentially identical. As cells divide, they self-arrange into complex 2D structures.

Clustering single timeframes by cell morphology and cell network morphology reveals a strong dependence on cell density. This is true for all YM and YMR parental and clonal populations. In both the 2 dimensional space resulting from a principal component dimensionality reduction and the space resulting from a UMAP clustering, low density cell networks span a relatively wide range, while high density networks cluster together (Supplementary fig 3). Networks derived from the same cell line, either YM or YMR, tend to cluster together regardless of cell density (Supplementary fig 3a, c), indicating that morphological differences exist between cell lines even at a relatively high cell density, when single cell morphology is highly impacted by contact with adjacent cells. Together, these observations suggest that an accurate morphological analysis needs to consider the entire growth trajectory of a cell lineage rather than just a single time point.

Temporal stability of SCC morphological profiles

To evaluate whether the observed morphological differences between clonal cell lineages are attributable to temporary fluctuations or to persistent, heritable differences, we performed additional experiments in which we selected a subset of three distinct clone lineages each from the set of YM and YMR clonal lineages (6 total) and continued our analysis through multiple (3-4) passage cycles over multiple weeks. These experiments showed that the derived biophysical characteristics, both at the single cell level and the cell network level, for each of these lineages were largely conserved across generations, with relatively minor drift (Supplementary fig 6A,B,C,D). Hierarchical clustering and UMAP dimensionality reduction of the clonal lineage generations across passage cycles grouped generations of the same clonal line together, evidence that real differences exist between distinct clonal lineages that persist over many generations (Supplementary fig 6E,F,G,H).

Comparisons with prior work

A number of similar previous works have investigated collective cell behavior in the context of tumor growth and invasion using a range of computational methods. In particular, [2] and [1] examine a physical phase transition between jammed and unjammed states that occurs in densely-packed cell populations. These studies also utilize single cell measurements and cell network-like representations in order to quantify behavior.

Our informatics approach, which incorporates deep learning segmentation, shares many similarities with these previous approaches. We similarly calculated parameters describing cell shape and neighboring order–analogous to measuring vertex degree of an adjacency graph. The only difference here is that in our method, adjacency is determined by contact at the cell boundary rather than cell centroid-to-centroid distance.

In this study, we did not look closely at collective cell phase transitions. The main reason for this is that we were primarily

© The Author 2022. Published by Oxford University Press. All rights reserved. For permissions, please e-mail: journals.permissions@oup.com

interested in the question of cell/cell population heterogeneity, so we designed our experiments and computational analysis to perform with a very high throughput, allowing us to analyze many cell lines simultaneously. As a result, we collect data with a relatively low temporal resolution (imaging once every 12 hours), which makes it difficult to precisely identify jamming/unjamming phase transitions that often depend on high resolution dynamic behavior.

Instead, we focused on deriving a broader set of morphological, local topological, and global topological features and combining these into an extensive biophysical signature. Clustering lineages based on all metrics simultaneously, as was done here, may help to differentiate between cell populations and identify heterogeneity.

At the same time, we remain interested in extending these methods to the study of biological phase transitions. A promising future direction would be to investigate how heterogeneity, both within and between cell populations, affects jamming and unjamming transitions. Here, metrics calculated using the methods described in these previous studies could feed into the informatics analysis to construct a more detailed biophysical picture.

Materials and Methods

Cell Culture

Prior to clonal lineage generation, YUMM1.7 (YM; Yale University Mouse Melanoma) and YUMMER1.7 (YMR; Yale University Mouse Melanoma Exposed to Radiation) cells were maintained at 37 °C and 5% CO2 in Dulbecco's Modified Eagle's Medium F12 (Life Technologies) supplemented with 10% fetal bovine serum (Catalog#16000044, Gibco), 1% Non-essential amino acids (Catalog#11140050, Gibco) and 1% penicillin–streptomycin (Life Technologies). These parental cell lines were a gift from the Bosenberg lab (YMR) and Miller-Jenson (YM) lab.

Single-cell Clone Generation

Individual cells were deposited into wells of 96 well plates using a FACSAria cell sorter. Cells were unstained and no selection criteria was applied, resulting in clonal populations that represent a random sampling from each of the parental populations. Clonal cells are expanded under the same conditions described above until they reach 90% confluency, at which point they are suspended using a 0.05% trypsin solution (Catalog#25200056, Gibco), and reseeded into a 6 well plate at a concentration of about 1300 cells/cm^2. These plates are cultured in the same media formulation and under the same conditions described above. Parental lineages are named YM P or YMR P for the YUMM parental and YUMMER parental lineages respectively. SCC lineages are named with either YM or YMR designating the originating cell line and a tag ranging from pre1 to 16.6 designating the single parental cell from which the clones are generated. All clones were isolated and expanded under identical conditions. All clones and parental lines were imaged under identical conditions.

2D Image Acquisition

Imaging for all clonal populations began 24 hours after the first seeding at low confluency in a 6 well plate and typically continued at 12 hour increments until 96 hours after initial seeding, or over one four day subculture cycle. All images used for the 2D morphological analysis were taken on a Leica DMi1 phase contrast microscope at 5x magnification with a 0.12 numerical aperture.

Image segmentation model training and verification

We implement the widely used Mask R-CNN architecture to perform instance segmentation on phase contrast images through the Detectron2 python library. This modular architecture is well suited for this task; it detects and segments individual cells, allowing us to extract both cell shape characteristics and population morphologies from a single segmentation output. Training images are input as $278^2\mu m^2$ (256^2px^2) tiles. Together, these include 4,946 single-cell annotations which were produced manually by a human annotator. We employ data augmentation, including brightness/contrast adjustment, mirroring, rotation, and cropping, applied sequentially, to artificially expand the number of distinct training examples. Training occurred for 10,000 epochs.

Quantification of large scale cell networks requires segmentation over larger areas than $278^2\mu m^2$. We achieve segmentation for larger images with a sliding 256^2px^2 window and a 128 px step size in x and y. As each region of the image is analyzed four times, the cumulative segmentation output from this process contains numerous duplicates. Pruning of these outputs is a crucial step to achieve accurate cell number and accurate segmentations. We implement a custom non-max-suppression algorithm that identifies duplicate masks based on the area overlap of the polygon masks and selects for objects with a higher classification confidence score. We additionally bias the algorithm to select for larger cells, ensuring that cells on the border of a prediction tile are not prioritized over cells in the center of a prediction tile. We note that this polygon non-max-suppression algorithm is computationally expensive compared to commonly used bounding box non-max-suppression algorithms for images with many object predictions. While this algorithm proved necessary for the images in this study, it may not be necessary for analyzing other images or cell types. Code for testing and training the segmentation model, as well as for the polygon and bounding box non-max-suppression algorithms is available at https://github.com/trevor-chan/Melanoma_NetworkMorphology.

Single-cell morphological quantification

Every cell in segmentation output is characterized by four morphological variables: area, perimeter, circularity (calculated as area/perimeter), and aspect ratio. For every image, we calculate the average and the variance of these properties for all cells in a single image.

Population morphological quantification

Cell population morphology describes 2D cell organization in one field of view. Cell networks are generated from segmented outputs. Every cell in the segmentation output corresponds to a node in the graph, and edges are constructed between adjacent cells, here defined as two cells with a minimum edge-to-edge distance less than 5 microns. From the resulting network, we derive a handful of relevant topological properties, including: average vertex degree, the average number of adjacent cells for each cell; number of components; max component mass, the proportion of all nodes in the network belonging the largest component; degree variance, the variance in vertex degree for all nodes in a network; and chromatic number, a measure of colorability that is closely associated

with graph degeneracy. Network generation, visualization, and topological property calculations utilize the NetworkX python package. We calculate fractal dimension from binarized images (produced from merging output masks of segmented images) using the box counting method.

Lineage characterization

As both single-cell morphology and population morphology are highly dependent on cell density, single time point snapshots are insufficient for robustly characterizing differences between clonal lineages. Images are acquired over multiple days as described above and morphological and topological quantities are derived for each. A lineage is therefore described by a collection of parameters in time and with respect to density. For this analysis, we ignore time and focus solely on cell density as the independent variable along which all morphological and topological quantities are compared. For each quantity with respect to density, we perform a polynomial regression and use the resulting fit to derive a set of general growth parameters that can be used to compare across lineages.

Unsupervised clustering and visualization

Clustering of SCC populations was accomplished using the Python SciKit-learn hierarchical clustering algorithm. Selection of the 6 morphological subclasses was performed manually by selecting an even cut across the population dendrogram at a user specified height. Resulting subclasses with a size less than 2 were excluded from further analysis, with the reason being outlier morphologies are both sufficiently rare and difficult to verify.

3D spheroid experiments

We followed a common spheroid generation protocol modeled after that described in (42). 1000 cells were deposited into each well of an agarose treated 96 well plate and centrifuged to pellet. Plates were cultured for 96 hours during which cells consolidated into spheroids and grew slightly. After 96 hours, spheroids were removed from the agarose plate and suspended in a collagen I solution with a final collagen concentration of 2 mg/ml. Collagen gels with a volume of 30 μl and containing one spheroid each were deposited onto the surface of a 24 well glass-bottomed plate. The collagen solution was allowed to polymerize at 37 °C, and the plate was periodically inverted during the initial gelation phase (10 minutes) to ensure spheroid suspension in the gel. 1 hour after the start of polymerization, media or media and drug solution was added. Imaging of spheroids in brightfield and reflectance occurred every 24 hours starting on day 0 immediately following the addition of media and ending on day 3 (t=72 hrs), after which cells were fixed and prepared for immunofluorescence imaging.

We fixed plates with 4% paraformaldehyde and for 30 minutes at 37 °C and washed plates with PBS. We then permeabilized with 0.1% Triton x-100 in PBS for 30 minutes at 37 °C and washed with PBS. We blocked with 1% BSA solution in PBS for 1 hour at 20 °C. We used Hoechst and phalloidin to fluorescently label nuclei and actin in 3D culture and image using a Leica SP-8 confocal microscope. All image stacks used for quantification are taken with a 10x magnification, 0.4 numerical aperture objective and a z-step size of 5 μm. Quantification of spheroid characteristics from the IF imaging data is explained in detail in the results section.

Supplementary Figures

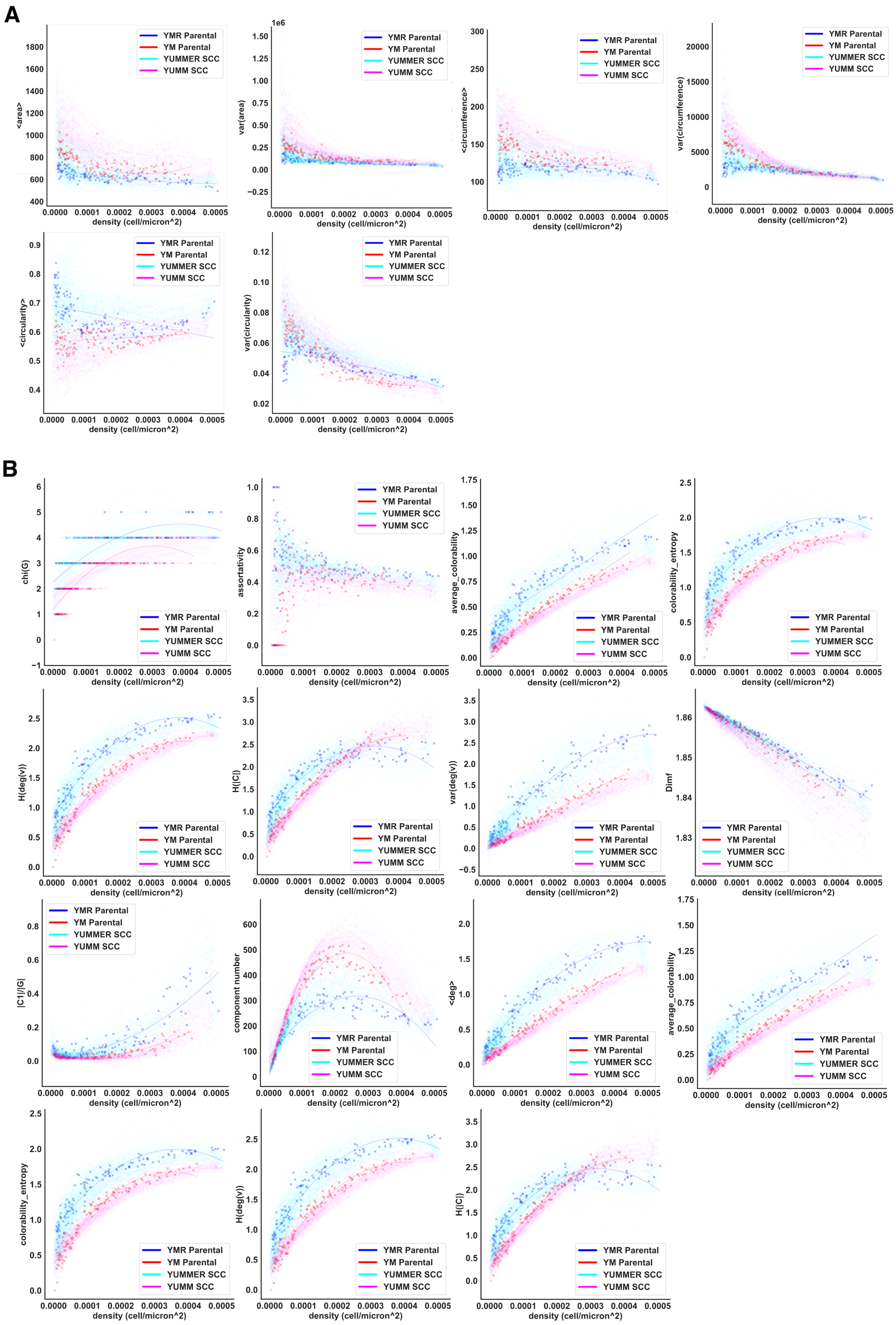

Supplementary Figure 1. SCC lineages are characterized by 22 total variables describing single cell morphology and cell network morphology over time.

(A,B) Single cell morphological variables **(A)** and cell-network variables **(B)** are shown plotted against cell density. Here YM and YMR parental and clonal lineages are plotted. Each point corresponds to a single image and contains data corresponding to the derived cell network and hundreds to thousands of cells. The collection of all data points from one clone over the course of a four day expansion period constitute a unique morphological progression. We use polynomial regression to fit curves to each clone morphological progression.

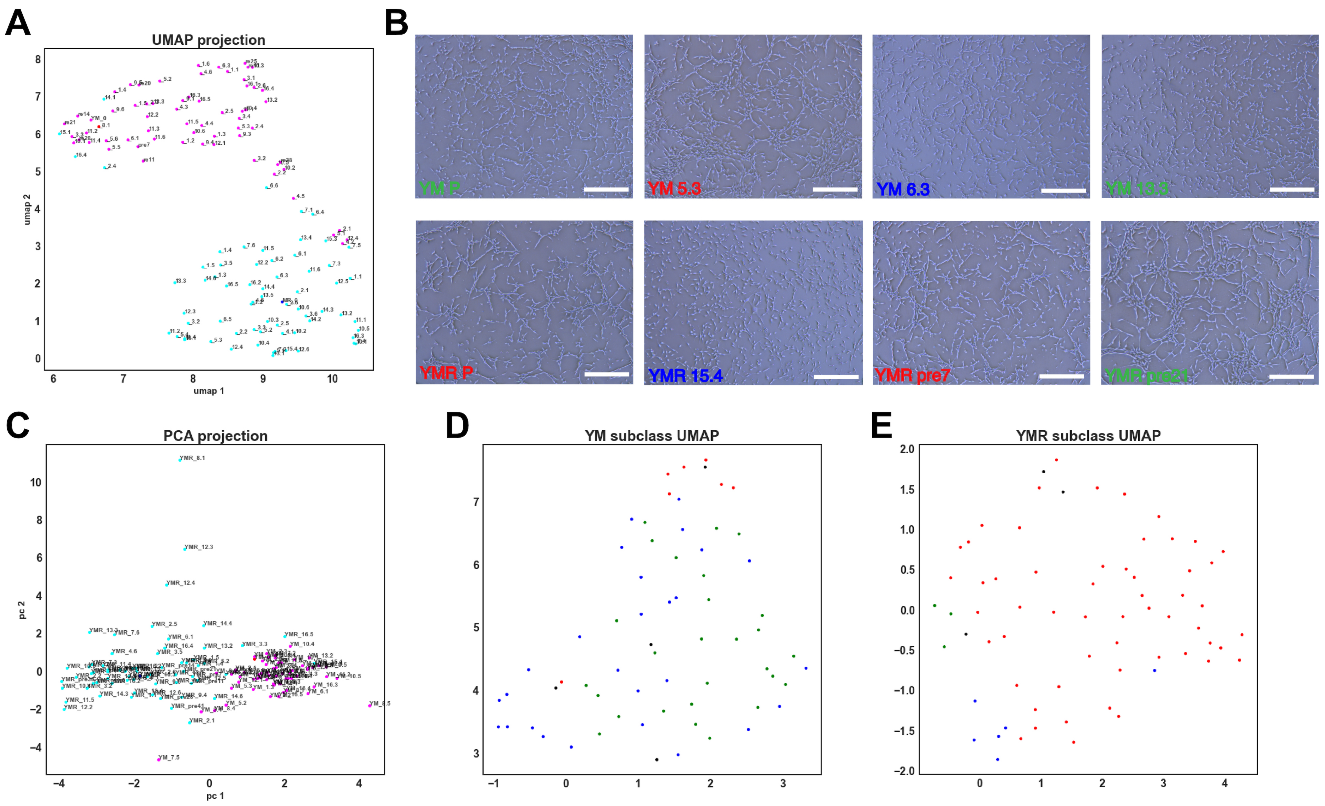

Supplementary Figure 2. Additional PCA and UMAP plotting of SCC lineages reiterates hierarchical clustering results. Representative images of cell clonal lineages are shown

(A) UMAP clustering of all clone lineages likewise distinguishes between YM derived and YMR derived clones and serves to corroborate the hierarchical clustering.

(B) Representative images taken from the parental lineages and one clone from each of the morphological subclasses show subtle differences in cell morphology and network structure at one point in time. See methods section for lineage naming convention. Images have a cell density of 0.000223+-0.00001 cells/μm^2 (1000 cell/FOV). Scale bar is 500 microns.

(C) Principal component analysis (2 component) of YM and YMR SCC populations, reiterating colocalization of populations originating from the same cell line. YM colored with magenta, YMR colored with cyan.

(D,E) UMAP clustering of YM populations **(D)** and YMR populations **(E)** with coloring according to morphological subclass obtained from hierarchical clustering results.

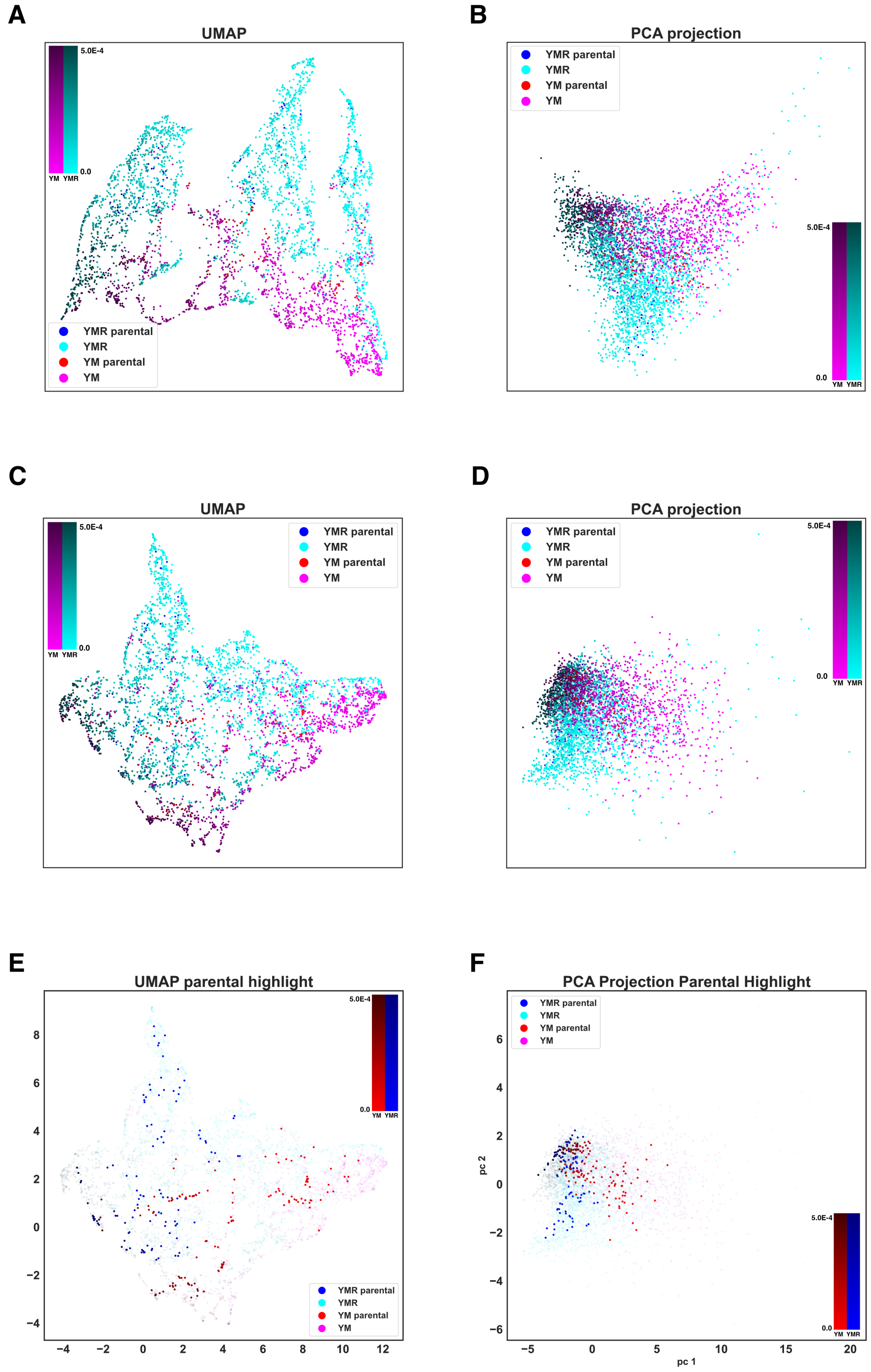

Supplementary Figure 3. Clustering of cell networks by single cell morphology reveals density and cell lineage dependent trends

(A,B) UMAP and PCA projection of cell networks from parental and SCC lineages from YM and YMR based on 22 cell morphology and network morphology variables. Points represent morphological measurements taken at a single time point. Density and cell number are not considered in the clustering and are depicted as a color gradient ranging from 0 cells/micron2 to 5E-4 cells/micron2. Clustering and PCA broadly shows colocalization of points with similar cell lines and densities. Differences between cell line morphology is initially stark at low cell density and fades as cells near high confluency.

(C,D) UMAP and PCA projection of cell networks from parental and SCC lineages from YM and YMR based on 12 variables describing only single-cell morphology. Clustering and figure settings are otherwise identical to plots A and B.

(E,F) UMAP and PCA projection of YM and YMR cell-networks with parental lineages highlighted. Clustering and projection is based on 12 variables describing only single-cell morphology.

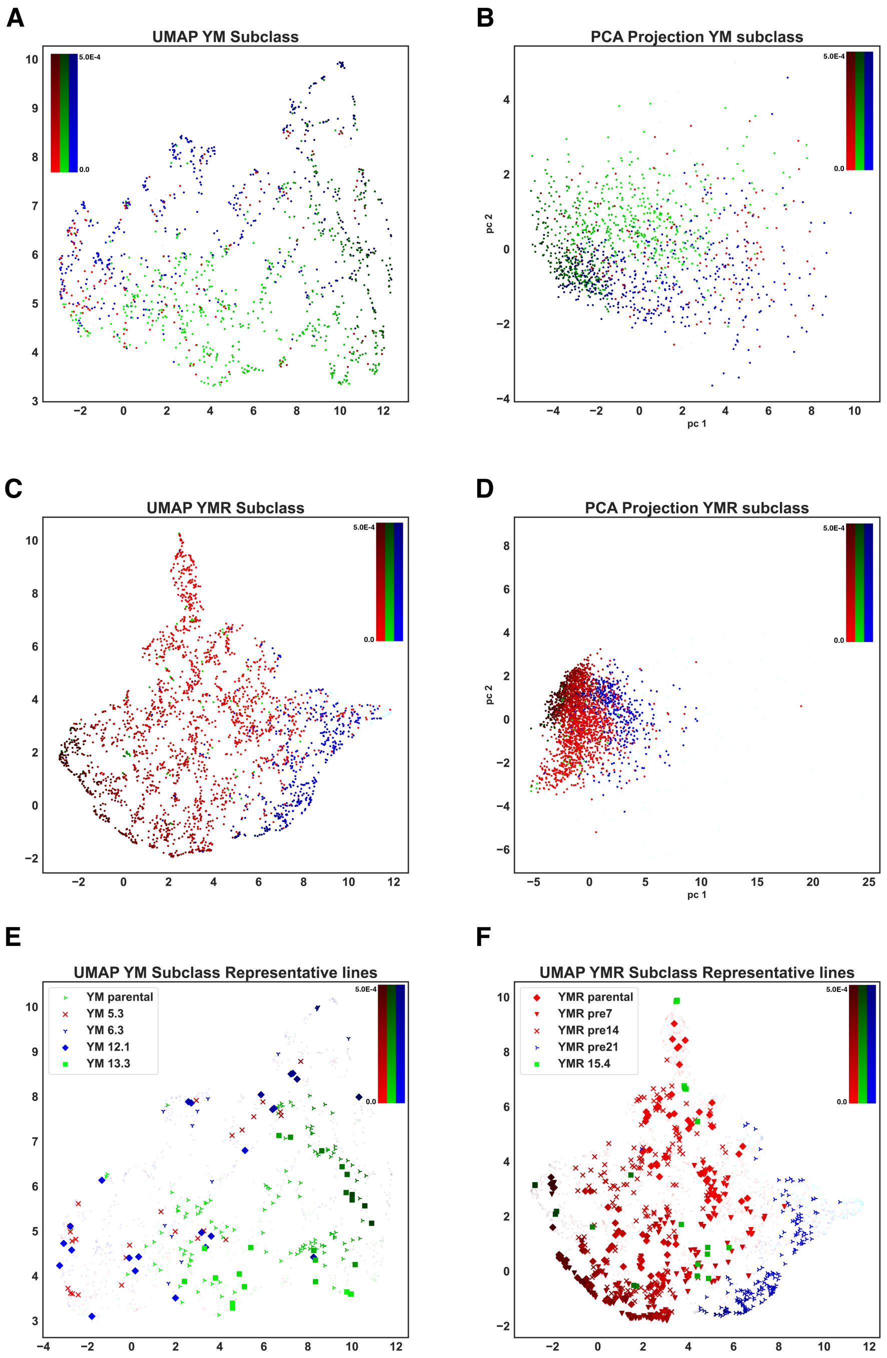

Supplementary Figure 4. Clustering of cell networks by single cell morphology reveals density and cell morphological subclass dependent trends.

(A,B,C,D) UMAP and PCA projection of YM and YMR cell-networks showing density and subclass projection. UMAP clustering and PCA projection of cell networks from parental and SCC lineages from YM and YMR based on 12 variables describing only single-cell morphology. Points represent morphological measurements taken at a single time point. Density and cell number are not considered in the clustering and are depicted as a color gradient ranging from 0 cells/μm^2 to 5E-4 cells/μm^2.

(E,F) UMAP clustering of YM and YMR populations showing morphological subclasses and highlighting representative SCC lineages used for downstream 3D analysis. Density is depicted as a color gradient ranging from 0 cells/μm^2 to 5E-4 cells/μm^2.

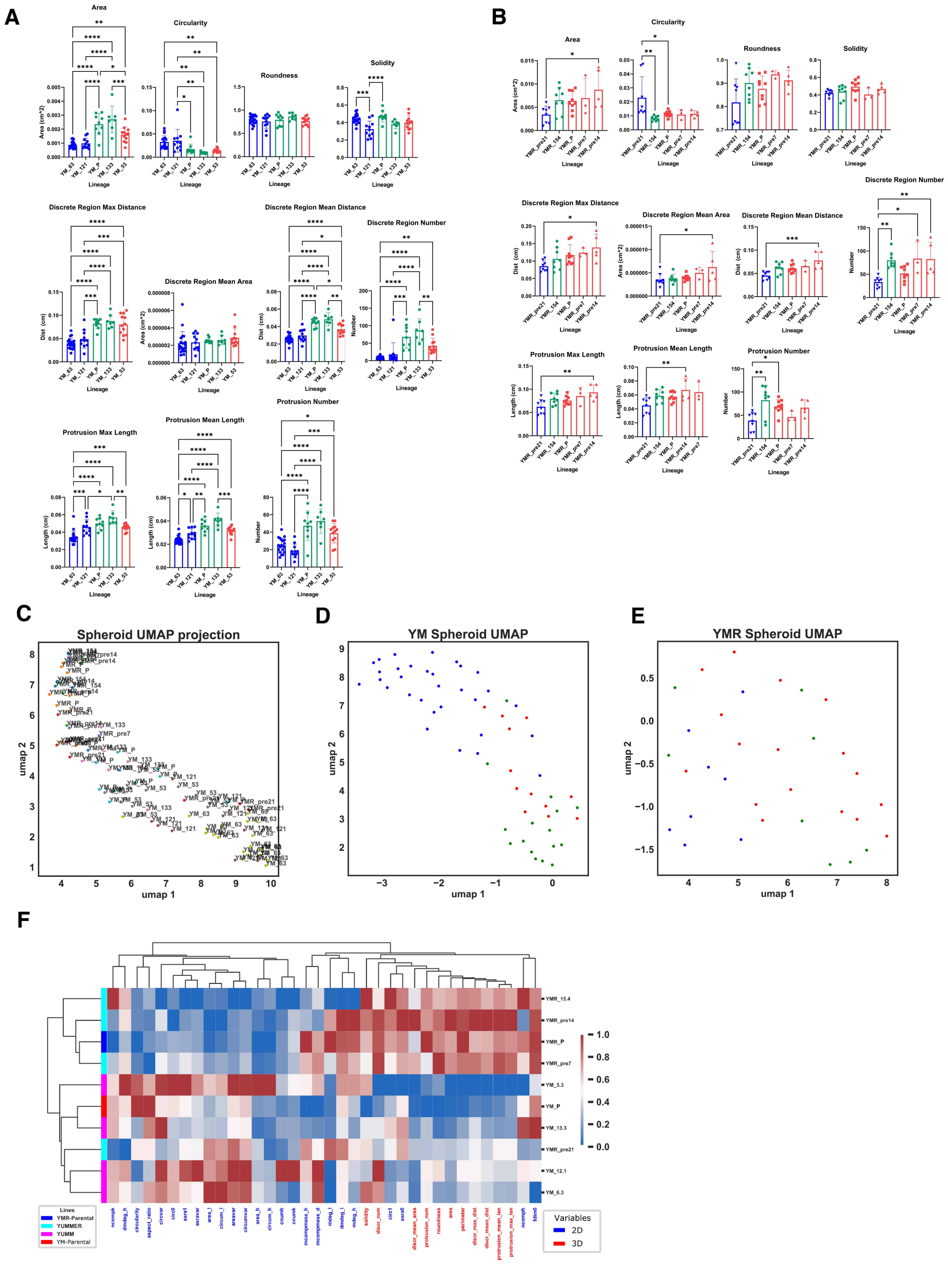

Supplementary Figure 5. 11 total derived spheroid variables describe morphology and invasion in a 3D spheroid assay. UMAP clustering of individual spheroids generates groups consistent with the 2D analysis based lineage clustering.

(A, B) A total of 11 variables are used to describe morphology and invasion characteristics in a 3D spheroid assay.

(C, D, E) UMAP clustering of spheroid data by lineage shows colocalization of spheroids with others from the same population **(C)** and from the same morphological subclass **(D, E)**.

(F) A hierarchical clustering of all 10 SCC lineages from both cell lines considering all 2D and 3D variables largely reiterates the findings of the 2D-only clustering and the 2D+3D-clustering on YM and YMR separately. Except for YMR pre21, lineages group with others of their cell line, and when present, others of their subclass. YMR pre21 falls across the cell line boundary on the dendrogram in 2D+3D, but it did not in 2D alone (fig 3a).

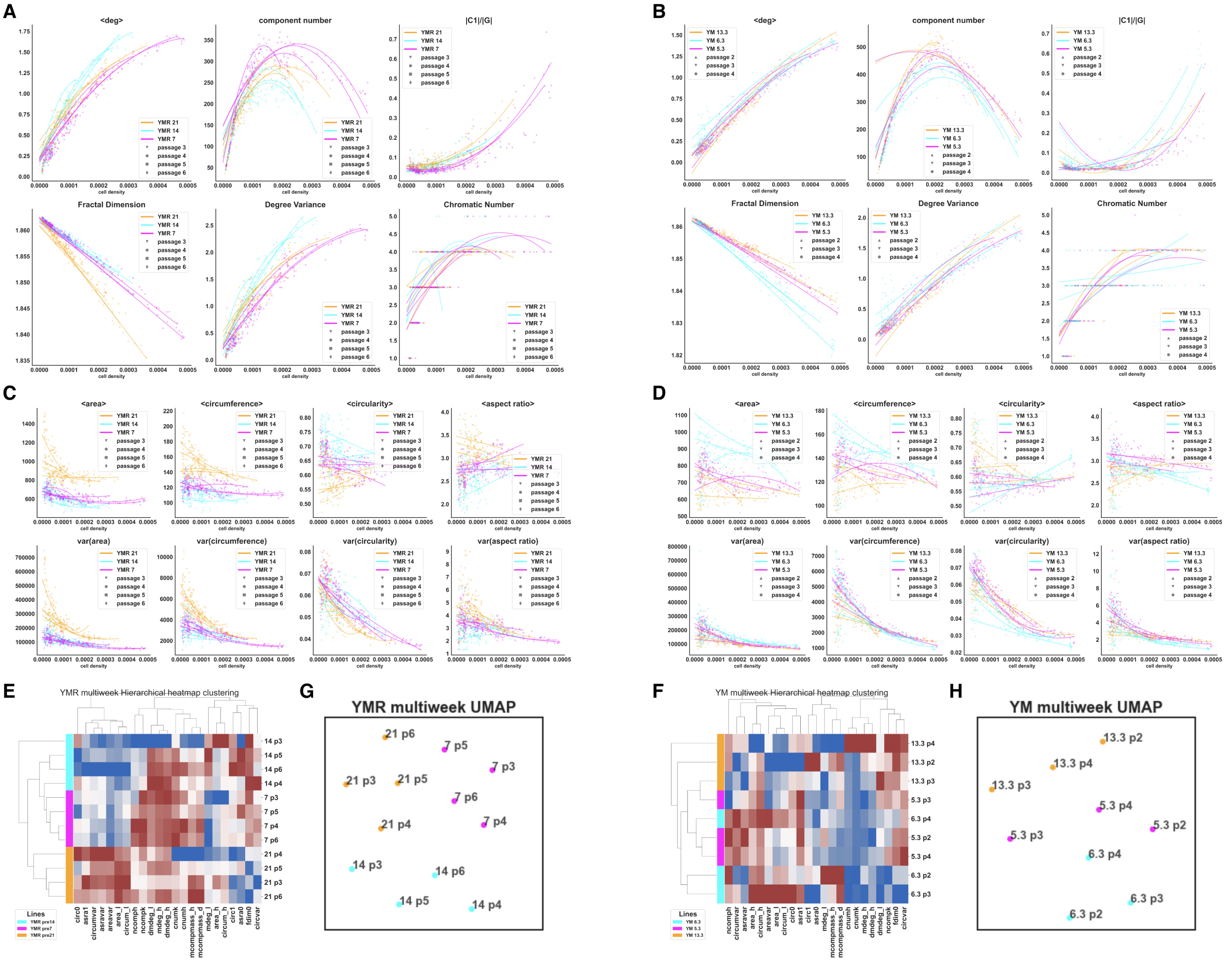

Supplementary Figure 6. SCC lineage morphological profiles remain stable and distinct over four cell passage cycles.

(A,B,C,D) Cell network topological variables for multiple passage cycles for YMR **(A)** and YM **(B)**, as well as single cell morphological variables for multiple passage cycles for YMR **(C)** and YM **(D)** representative lineages are shown plotted against cell density. For each repeat passage cycle, points are plotted and a new curve is fit using polynomial regression. We show overall consistent morphodynamic behavior with little drift over the multiple week-long observation period.

(E,F,G,H) Separate passage cycles of multiple lineages are grouped using a hierarchical clustering algorithm **(E,F)** and UMAP **(G,H)**. We show that passage cycles of the same clonal lineage cluster together, further evidence that drift in cell morphological properties is minor with respect to differences between distinct clonal lineages.

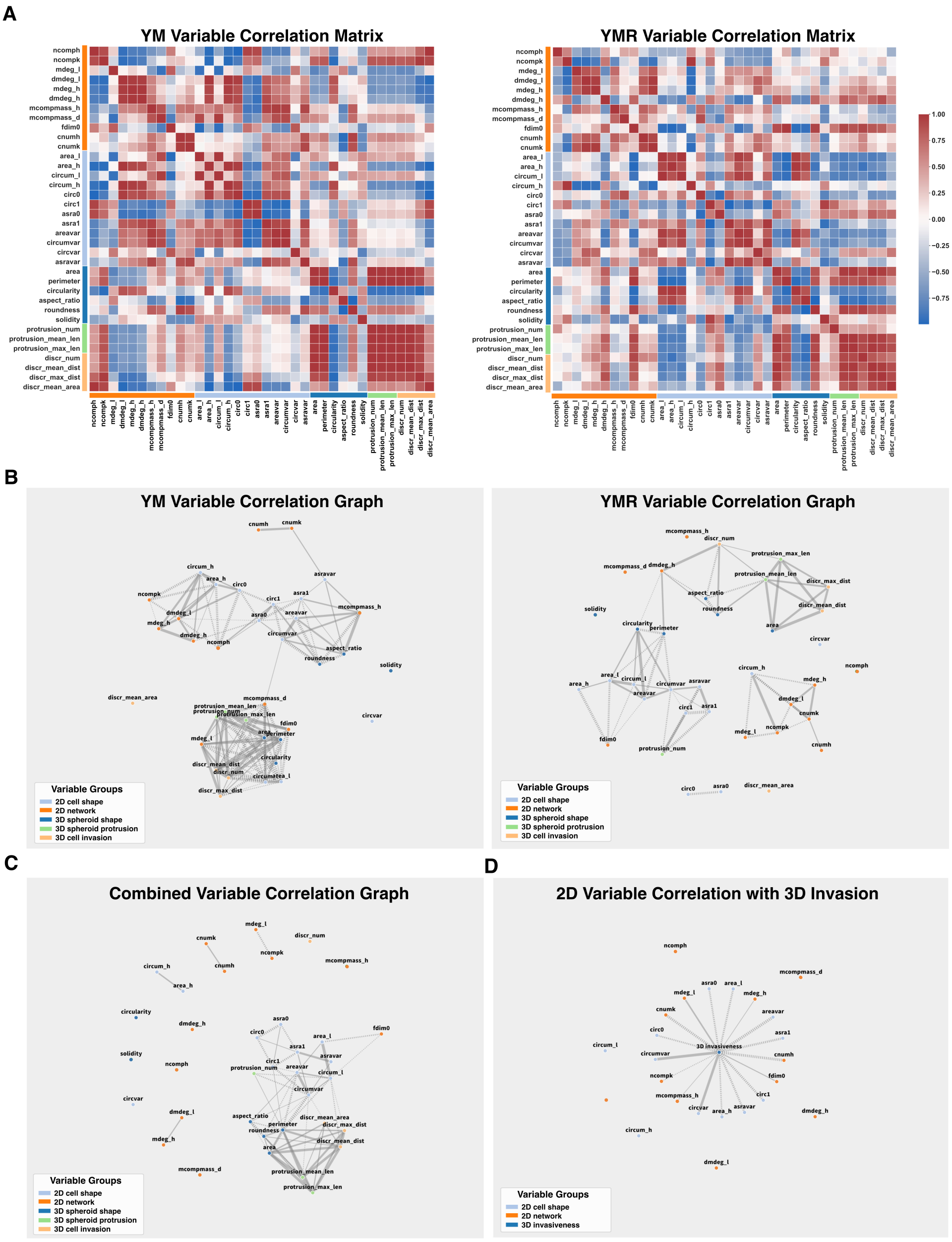

Supplementary Figure 7. As additional analysis, we repeat our calculations of variable correlations using the Pearson's correlation test. We note that this test assumes the derived morphological variables are linearly distributed. For equivalent results under a relaxation of the linear assumption, refer to Figure 5 of the main text, which uses a Spearman's rank correlation test.

(A) Correlation matrices show numerous strong positive and negative correlations between morphological variables. Correlations are common between variables describing behavior at the same scale (single cell scale, cell network scale, cell spheroid scale) but also exist between variables describing behavior across scales.

(B,C,D) A variable correlation graph provides an alternate visualization of variable relationships. Here, nodes of the graph represent variables and are colored according to the scale of behavior each describes-the variable group. Solid edges represent positive correlations and dashed edges represent negative correlations. Thicker lines represent stronger (larger magnitude) correlations.

(B) Variable correlation graphs depict YM and YMR SCC populations separately. Correlation matrices Edges represent a variable to variable Pearson correlation coefficient magnitude greater than 0.9.

(C) Variable correlation graph depicts YM and YMR SCC populations together. Correlation matrices Edges represent a variable to variable Pearson correlation coefficient magnitude greater than 0.9.

(D) Variable correlation graph depicts the correlations between 2D variables and a single variable for 3D invasiveness only. (Relations between 2D variables are not drawn) This graph includes SCC populations from both the YM and YMR cell lines. Correlation matrices Edges represent a variable to variable Pearson correlation coefficient magnitude greater than 0.3.

Supplementary Videos

All videos available at https://drive.google.com/drive/folders/132u1dtk1Loj7qD8gi9-t6aFuCllT6uWB?usp=sharing.

SI Video 1: A representative YM parental spheroid is visualized in 3d. F-actin is labeled as magenta and DNA is labeled as cyan. https://drive.google.com/file/d/1Zcoyhz52OnRvtx2g0tO_kO3RAc4-GWlV/view?usp=sharing

SI Video 2: A representative YM 5.3 spheroid is visualized in 3d. F-actin is labeled as magenta and DNA is labeled as cyan. https://drive.google.com/file/d/1SW_8dcCjw6mI3z-28SbbGbUyZe6ODH8F/view?usp=sharing

SI Video 3: A representative YM 6.3 spheroid is visualized in 3d. F-actin is labeled as magenta and DNA is labeled as cyan. https://drive.google.com/file/d/1uMKxOyAuTrjzd3hSWLGcNKOgrF4kDZlm/view?usp=sharing

SI Video 4: A representative YM 12.1 spheroid is visualized in 3d. F-actin is labeled as magenta and DNA is labeled as cyan. https://drive.google.com/file/d/16dTDl85wtaoWA2WDC5P7WIshwMNMRAOY/view?usp=sharing

SI Video 5: A representative YM 13.3 spheroid is visualized in 3d. F-actin is labeled as magenta and DNA is labeled as cyan. https://drive.google.com/file/d/1Pjeh1RqZyUS02w4-HNm7dD9Ka8iyAk4L/view?usp=sharing

SI Video 6: A representative YMR parental spheroid is visualized in 3d. F-actin is labeled as magenta and DNA is labeled as cyan. https://drive.google.com/file/d/1vp7gkHjWO-q80xlvsCNjtPQ-q-mjPCsj/view?usp=sharing

SI Video 7: A representative YMR pre7 spheroid is visualized in 3d. F-actin is labeled as magenta and DNA is labeled as cyan. https://drive.google.com/file/d/1gNLWAB4qc3z4Dycf7x-aOKtNXsHCOK1s/view?usp=sharing

SI Video 8: A representative YMR 15.4 spheroid is visualized in 3d. F-actin is labeled as magenta and DNA is labeled as cyan. https://drive.google.com/file/d/1ocVsKcMatJa1Vo4WvMrb5EmA6VTU15kK/view?usp=sharing

SI Video 9: A representative YMR pre14 spheroid is visualized in 3d. F-actin is labeled as magenta and DNA is labeled as cyan. https://drive.google.com/file/d/1izXwFaMjcBpOylW9RrUtHY5TvGgRlHVy/view?usp=sharing

SI Video 10: A representative YMR pre21 spheroid is visualized in 3d. F-actin is labeled as magenta and DNA is labeled as cyan. https://drive.google.com/file/d/13DdGPvpTcT1gqvayMu4gBvh_FNuG-w5i/view?usp=sharing

Supplemental References

References

1. Wenying Kang, Jacopo Ferruzzi, Catalina-Paula Spatarelu, Yu Long Han, Yasha Sharma, Stephan A Koehler, Jennifer A Mitchel, Adil Khan, James P Butler, Darren Roblyer, et al. A novel jamming phase diagram links tumor invasion to non-equilibrium phase separation. *Iscience*, 24(11):103252, 2021.
2. Haiqian Yang, Adrian F Pegoraro, Yulong Han, Wenhui Tang, Rohan Abeyaratne, Dapeng Bi, and Ming Guo. Configurational fingerprints of multicellular living systems. *Proceedings of the National Academy of Sciences*, 118(44):e2109168118, 2021.
